# Sera total oxidant/antioxidant status in lung cancer patients

**DOI:** 10.1097/MD.0000000000017179

**Published:** 2019-09-13

**Authors:** Miao Xiang, Jiafu Feng, Lidan Geng, Yuwei Yang, Chunmei Dai, Jie Li, Yao Liao, Dong Wang, Xiao-Bo Du

**Affiliations:** a Department of Oncology; b Department of Clinical Laboratory, Mianyang Central Hospital, Mianyang; c Department of Oncology, Affiliated Hospital of North Sichuan Medical College, Nan Chong; d Department of Surgery, Mianyang Central Hospital, Mianyang, Sichuan, P.R. China.

**Keywords:** lung cancer, oxidative stress, oxidative stress index, total antioxidant status, total oxidant status

## Abstract

We investigated oxidative stress parameters in the sera of patients with lung cancer and healthy individuals to evaluate their correlations with lung cancer.

Ninety-four lung cancer patients and 64 healthy controls were enrolled after obtaining informed consent. Their sera oxidative stress parameters were measured.

Total antioxidant status (TAS), total oxidant status (TOS), and oxidative stress index (OSI) were significantly different between patients and healthy groups (all *P* < .001). TAS gradually decreased and TOS and OSI gradually increased from stage I to III, but it did not reach statistical significance (all *P* > .05). TAS and OSI were significantly different between the nonsmoking and smoking groups, radiotherapy and without radiotherapy groups, chemotherapy and without chemotherapy groups (*P* < .05), but not TOS (*P* > .05). In a receiver operating characteristic curve analysis comparing patients with lung cancer with healthy controls, the Youden indices of TOS, TAS, and OSI were 0.541, 0.532, and 1, respectively.

The oxidative stress may be correlation with lung cancer staging. Smoking, surgery, radiotherapy, and chemotherapy showed correlation with parts oxidative stress parameters.

## 1 Introduction

Lung cancer is the most prevalent cancer among both men and women, and it is the leading cause of cancer-related deaths in the United States.^[1]^ In total, 1.8 million new lung cancer cases and 1.6 million lung cancer deaths were reported in 2012 worldwide, accounting for approximately 19% of all cancer-related deaths.^[2,3]^ It is also the most common type of cancer and the leading cause of cancer-related deaths in China.^[4]^ Lung cancer poses serious health problems. However, its etiology is unclear. A better understanding of the etiology can help to monitor and to treat this condition.

Free radicals and reactive oxygen species (ROS) are found in all aerobic organisms. If free radical and ROS levels are elevated, this may cause oxidative damage to the body.^[5]^ The formation of ROS and the clearance of antioxidants constitute the body's peroxidation–antioxidation system, which is in dynamic equilibrium under normal physiological conditions.^[6]^ An imbalance in the system leads to oxidative stress, which has been implicated in the pathogenesis of cancer. Oxidative stress plays a role in carcinogenesis and the development of lung cancer. In human lung cancer cells, higher levels of DNA modifications were associated with lower glutathione peroxidase activity and reduced gene expression of glutathione peroxidase in normal bronchial epithelial cells, and this has been considered to be a risk factor for the development of lung cancer in cigarette smokers.^[7,8]^ Nuclear factor erythroid 2 related factor 2 (Nrf2) activates cellular rescue pathways against oxidative injury. However, the prolonged activation of Nrf2 favors the progression of several types of cancer, including lung cancer,^[9]^ and elevated levels of Nrf2 promote the production of antioxidants, such as glutathione, and maintain the redox balance and the transcription of several genes involved in the proliferation of cancer cells.^[10]^ Oxidative stress-induced tumor initiation or progression may result from the overproduction of ROS by the members of the NADPH oxidase (NOX) family.^[11]^ NADPH oxidase 4 (NOX4) is deregulated in various cancers and is involved in cancer proliferation and metastasis, and the positive feedback loop between NOX4 and PI3K/Akt signaling contributes to nonsmall cell lung cancer progression.^[12]^ NOX activity and expression are associated with the tumorigenesis of lung cancer, and the inhibition of NOX function or mRNA expression significantly prevents the development and invasion of lung cancer.^[13]^

Currently, some studies have shown an increase in lipid peroxidation and reduction in total antioxidant activity due to smoking, which is associated with tumorigenesis.^[14]^ Concurrent with our research, an association between thyroid cancer,^[15]^ prostate cancer,^[16]^ and cervical cancer^[17]^ as well as oxidative stress was found. An imbalance between oxidation and antioxidation was previously observed in patients with lung cancer.^[18]^ A single measurement index could not effectively reflect the level of oxidative stress in the serum. Therefore, total oxidant status (TOS), with hydrogen peroxide and lipid hydroperoxide as the main components, is used to measure the overall oxidation state and the total antioxidant status (TAS), which includes the antioxidative effects of bilirubin, uric acid, vitamin C, polyphenols, and proteins, and the combined activities of all antioxidants.^[19,20]^

Considering the abovementioned data, this study aimed to investigate the relationship between oxidative stress status and lung cancer. Moreover, the serum levels of TAS, TOS, and OSI in patients with lung cancer were compared with those of healthy controls, and the factors associated with such condition were assessed.

## 2 Materials and methods

### 2.1 Objects

Blood samples from 94 patients with lung cancer and 64 healthy people were obtained between 2014 and 2015. Patients were diagnosed by clinical, imaging, and pathological examinations. Essential information such as gender, age, smoking history, history of other chronic diseases, pathological type of cancer, its clinical stage, radiation, chemotherapy, and surgery of the patients was recorded. The patients with lung cancer were divided into stages I to IV according to AJCC (7th edition) of tumor node metastasis (TNM).^[21]^ For healthy controls, the inclusion criteria included healthy individuals aged 18 to 70 years and those with no history of cancer and medication use within a month prior to enrollment or during the study. Healthy individuals or patients with other diseases, such as hypoglycemia, malnutrition, gout, liver diseases, rheumatoid arthritis, diabetes mellitus, lipid disturbances, and thyroid disease, were excluded because these diseases could be confounding variables.

The study was ratified by the medical ethics committee of our Hospital (number: 2013042). Before obtaining blood, patients and healthy or close relatives signed informed consent and agreed to use it for research purposes.

### 2.2 Blood specimens

Patients and healthy people were fasted for more 8 hours and peripheral venous blood samples were collected into the BD Vacutainer tube (anti-coagulation tube with inert separation gel, free of preservatives) using the disposable venous blood collection needle. Approximately, 3 to 4 mL blood was collected. After 1 hour, but within 2 hours, blood samples were centrifuged at 3000 rpm for 15 minutes. The oxidative stress markers were measured within 24 hours. Blood specimens were laid in −70 °C if more than 24 hours were required.

### 2.3 Detection method

Serum TOS was detected using xylenol orange method.^[22]^ TAS was detected by ABTS using a colorimetric assay, which is based on the antioxidant capacity of the samples. ABTS was used as a chromogenic agent. OSI is the ratio of TOS to TAS, which is calculated as (arbitrary units) = [(TOS, μmolH_2_O_2_equialent./L)/(TAS, μmol Trolox equiv./L) × 100].^[23]^

### 2.4 Statistical analysis

We performed statistical analysis by SPSS 19.0 software. All data conformed to normal distribution. Kolmogorov–Smirnov test was used to detect whether the results of the measured oxidative stress parameters (TOS, TAS, and OSI) were in normal distribution. If it is a normal distribution, 2 groups using *t* test, and multiple sets using ANOVA analysis were presented as F values. *P* values <.05 were defined as statistically significant. We used MedCalc statistical software (MedCalc, Mariakerke, Belgium) to draw the receiver operating characteristic (ROC) curves.

## 3 Results

Demographics and clinical data of both groups A and B are summarized in Table 1. The levels of OSI, TAS, and TOS were compared between groups A and B and the results are given in Table 2. The Kolmogorov–Smirnov test showed that these parameters are normally distributed. The serum TOS and OSI in patients with lung cancer was significantly higher than in controls (*P* < .001). The serum TAS in healthy controls was significantly higher than in the patients with lung cancer (*P* < .05).

**Table 1:**
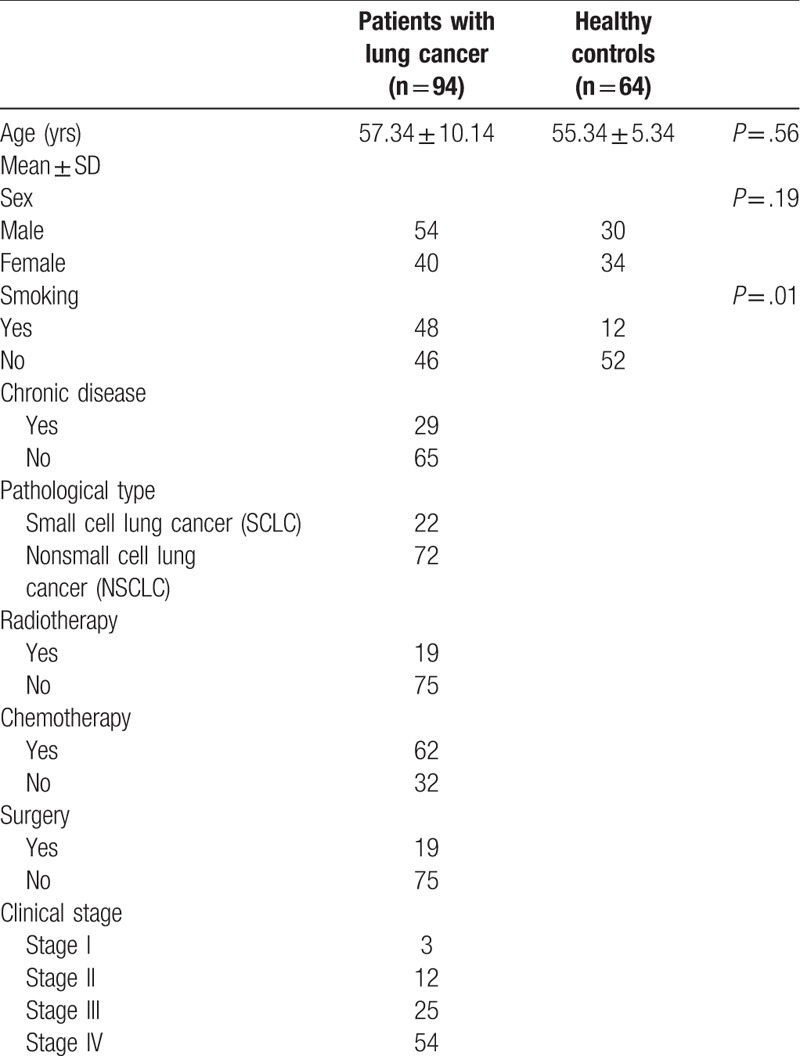
Demographics and clinical data of patients and healthy controls.

**Table 2:**
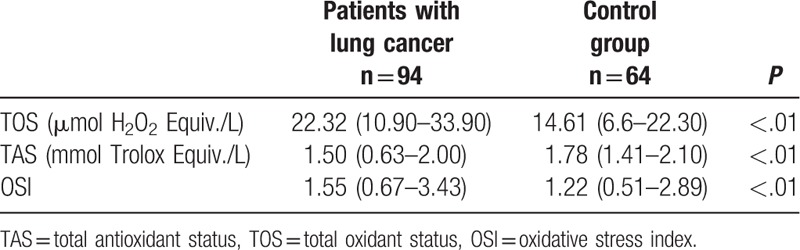
Comparison of TOS, TAS, and OSI levels between lung cancer group and healthy control group.

The results of one-way ANOVA analysis (Table 3) showed that the mean value of TOS and OSI gradually increased and that of TAS gradually decreased from stages I to III. But it did not reach statistical significance (*P* > .05). There were no significant differences in OSI, TOS, and TAS between 2 pathological type (*P* > .05). There were significant differences in OSI and TAS between the nonsmoking and smoking groups, radiotherapy and without radiotherapy groups, chemotherapy and without chemotherapy groups (*P* < .05), but not TOS (*P* > .05). Only OSI was significantly different between the surgery and no operated groups (Table 3).

**Table 3:**
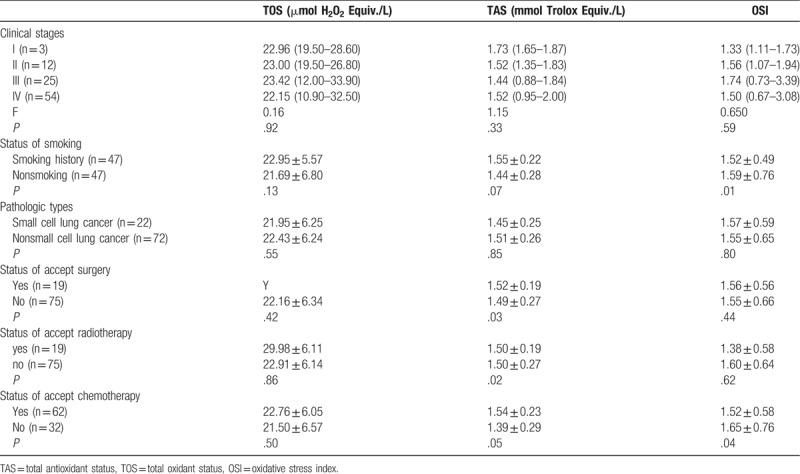
Serum level of oxidative stress parameters of in patients with lung cancer.

**Figure 1:**
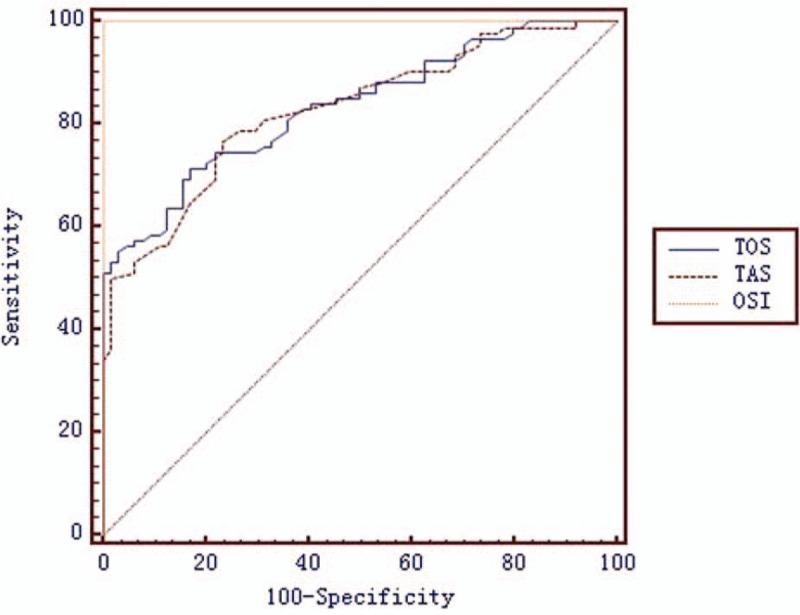
The receiver operating characteristic (ROC) curve analyses of the oxidative stress parameters in lung cancer diagnosis. AUC = area under the curve.

The diagnostic significance of TAS, TOS, and OSI for lung cancer was analyzed using ROC curve analysis (Fig. 1). The area under the curve (AUC) was calculated and the cutoff values, specificity, *P* values, sensitivity, and Youden indices (YIs), are detailed in Table 4. Compared with healthy controls, the AUC values of TOS, TAS, and OSI were 0.834 (0.767–0.889), 0.828 (0.760–0.883), and 1.000 (0.977–1.000), respectively, and the YIs were 0.541, 0.532, and 1, respectively. The AUC values for all parameters were >0.8. The optimal cutoff value of serum TOS,TAS, and OSI has a good sensitivity (71.3%,76.6%, and 100%, respectively) and specificity (82.8%,76.6%, and 100%, respectively) for distinguishing between patients with lung cancer and healthy controls (Table 4).

**Table 4:**
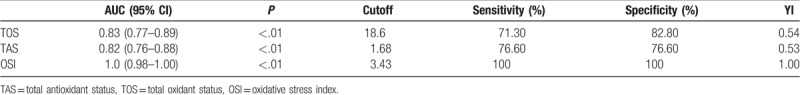
Evaluation of oxidative stress parameters in the diagnosis of lung cancer.

## 4 Discussion

Several studies have confirmed that there exist differences between patients with cancer and healthy controls in terms of oxidative stress parameters. Feng et al ^[24]^ have measured the serum oxidative stress parameters of individuals with breast cancer and benign breast tumors and healthy participants. The serum levels of TOS and OSI were higher in patients with benign and malignant tumors than in healthy controls; however, TAS levels were significantly lower in patients with benign and malignant tumors than in healthy controls. The same results were found in thyroid cancer, colorectal cancer, esophageal cancer.^[15,25,26]^ The present study showed that patients with lung cancer had higher serum TOS and OSI levels but lower TAS levels than the healthy controls. Peddireddy et al ^[27]^ have reported that a higher rate of oxidative stress might play a role in the pathogenesis of lung cancer, as evidenced by a failure in the oxidant/antioxidant balance in favor of lipid peroxidation and DNA damage. The previous study has shown a significant difference in advanced oxidation protein product levels between patients with lung cancer and healthy controls.^[28,29]^ However, the methods used in such studies were different from those in the present study.

The relationship between oxidative stress parameters and clinical stage in patients with breast cancer, prostate cancer, esophageal cancer, and colorectal cancer was assessed.^[25,26,30,31]^ In our study, TOS and OSI levels increased with increasing clinical stage, particularly in stages I to III; in stage IV, the TOS and OSI levels decreased and the TAS level increased. However, no significant difference was observed in TOS, OSI, and TAS levels in individuals with different clinical stages of lung cancer. The causes of this pattern are not fully elucidated and may be related to chance, insufficient number of few cases, or reduction of oxidative stress in stage IV cancer.

In the present study, TAS and OSI levels, but not TOS level, were significantly different between the nonsmoking and smoking groups. However, the relationship between cancer and oxidative stress is controversial. In breast cancer and colorectal cancer, a significant increase in oxidative stress have been observed in patients with a history of smoking.^[25,32]^ However, smoking history was not correlated to the parameters of oxidative stress or oxidative stress-modifying genes in pancreatic cancer and colorectal cancer.^[26,33,34]^

Chemotherapy and radiotherapy can change the levels of oxidative stress and antioxidants in patients with cancer. Taherkhani et al ^[35]^ have shown that a concurrent significant increase in malondialdehyde levels and a significant decrease in TAS levels were observed after 3 cycles of adriamycin and cytoxan chemotherapy in patients with breast cancer. However, Mohan et al ^[36]^ and Krawczyk et al ^[37]^ have observed no oxidative stress parameters change was observed after chemotherapy in nonsmall cell lung cancer (NSCLC). And the oxidative stress parameters have the different changes after radiotherapy in different cancer.^[38,39]^ Our study has shown that chemotherapy and radiotherapy can increase the levels of oxidative stress and antioxidants. However, the antioxidant level was higher than the oxidative stress level. Bukan et al ^[40]^ have indicated that open surgery could lead to oxidative stress, but another study showed that serum oxidative stress parameters of preoperative and postoperative had no significant differences^[41]^ In our study, only OSI level was significantly different between the surgery and no surgery groups. Only 19 patients underwent surgery, which may have resulted in the differences in the previous studies.

Oxidative stress parameters can potentially facilitate the development of biomarkers of cancers, including prostate cancer and esophageal cancers.^[26,31]^ In the present study, based on the ROC curve, the optimal cutoff values of serum TOS and OSI had good sensitivity and specificity for lung cancer. This demonstrates that TAS and OSI are potential diagnostic biomarkers that can be used to distinguish patients with lung cancer from healthy individuals. Our research is consistent with previous studies.^[42]^ However, additional research is necessary to confirm these findings.

The present study had some limitations. First, the sample only included a low number of patients with different stages of lung cancer, particularly stages I and II, and this leads to conflicting results about the relationship between oxidative stress parameters and lung cancer stages. Second, healthy people or patients with malnutrition were excluded, and we cannot validate whether the nutritional status of both groups is similar, which might have affected the results of oxidative stress parameters.

## 5 Conclusion

Serum TOS, TAS, and OSI were significantly different between patients with lung cancer and healthy controls. The oxidative stress may be correlation with lung cancer staging, but additional study is needed to affirm it. Smoking status, surgery, radiotherapy, and chemotherapy showed associated with parts oxidative stress parameters.

## Author contributions

**Conceptualization:** Dong Wang, Xiaobo Du.

**Data curation:** Miao Xiang, Jiafu Feng, Lidan Geng, Yuwei Yang, Chunmei Dai, Jie Li, Yao Liao.

**Formal analysis:** Miao Xiang, Jiafu Feng.

**Investigation:** Miao Xiang, Jiafu Feng, Lidan Geng, Jie Li, Yao Liao.

**Methodology:** Jiafu Feng, Yuwei Yang, Chunmei Dai.

**Project administration:** Jiafu Feng, Dong Wang.

**Supervision:** Dong Wang, Xiaobo Du.

**Writing – original draft:** Miao Xiang.

**Writing – review & editing:** Xiaobo Du.
